# Genome assembly of the pioneer species *Plantago major* L. (Plantaginaceae) provides insight into its global distribution and adaptation to metal-contaminated soil

**DOI:** 10.1093/dnares/dsad013

**Published:** 2023-05-25

**Authors:** Shanwu Lyu, Qiming Mei, Hui Liu, Baosheng Wang, Jun Wang, Hans Lambers, Zhengfeng Wang, Bin Dong, Zhanfeng Liu, Shulin Deng

**Affiliations:** Guangdong Provincial Key Laboratory of Applied Botany, South China Botanical Garden, Guangzhou 510650, China; Key Laboratory of South China Agricultural Plant Molecular Analysis and Genetic Improvement South China Botanical Garden, Chinese Academy of Sciences, Guangzhou 510650, China; Guangdong Provincial Key Laboratory of Applied Botany, South China Botanical Garden, Guangzhou 510650, China; Key Laboratory of Vegetation Restoration and Management of Degraded Ecosystems & CAS Engineering Laboratory for Vegetation Ecosystem Restoration on Islands and Coastal Zones, South China Botanical Garden, Chinese Academy of Sciences, Guangzhou 510650, China; Guangdong Provincial Key Laboratory of Applied Botany, South China Botanical Garden, Guangzhou 510650, China; Key Laboratory of Vegetation Restoration and Management of Degraded Ecosystems & CAS Engineering Laboratory for Vegetation Ecosystem Restoration on Islands and Coastal Zones, South China Botanical Garden, Chinese Academy of Sciences, Guangzhou 510650, China; Guangdong Provincial Key Laboratory of Applied Botany, South China Botanical Garden, Guangzhou 510650, China; Key Laboratory of Plant Resources Conservation and Sustainable Utilization, South China Botanical Garden, Chinese Academy of Sciences, Guangzhou 510650, China; Guangdong Provincial Key Laboratory of Applied Botany, South China Botanical Garden, Guangzhou 510650, China; Key Laboratory of Vegetation Restoration and Management of Degraded Ecosystems & CAS Engineering Laboratory for Vegetation Ecosystem Restoration on Islands and Coastal Zones, South China Botanical Garden, Chinese Academy of Sciences, Guangzhou 510650, China; School of Biological Sciences, University of Western Australia, Perth, WA 6009, Australia; Guangdong Provincial Key Laboratory of Applied Botany, South China Botanical Garden, Guangzhou 510650, China; Key Laboratory of Vegetation Restoration and Management of Degraded Ecosystems & CAS Engineering Laboratory for Vegetation Ecosystem Restoration on Islands and Coastal Zones, South China Botanical Garden, Chinese Academy of Sciences, Guangzhou 510650, China; Guangdong Agriculture Industry Business Polytechnic, Guangzhou 510507, China; Guangdong Provincial Key Laboratory of Applied Botany, South China Botanical Garden, Guangzhou 510650, China; Key Laboratory of Vegetation Restoration and Management of Degraded Ecosystems & CAS Engineering Laboratory for Vegetation Ecosystem Restoration on Islands and Coastal Zones, South China Botanical Garden, Chinese Academy of Sciences, Guangzhou 510650, China; Guangdong Provincial Key Laboratory of Applied Botany, South China Botanical Garden, Guangzhou 510650, China; Key Laboratory of South China Agricultural Plant Molecular Analysis and Genetic Improvement South China Botanical Garden, Chinese Academy of Sciences, Guangzhou 510650, China

**Keywords:** *Plantago major*, genome assembly, global distribution, plant specialized metabolites, adaptation

## Abstract

*Plantago* is a major genus belonging to the Plantaginaceae family and is used in herbal medicine, functional food, and pastures. Several *Plantago* species are also characterized by their global distribution, but the mechanism underpinning this is not known. Here, we present a high-quality, chromosome-level genome assembly of *Plantago major* L., a species of *Plantago*, by incorporating Oxford Nanopore sequencing and Hi-C technologies. The genome assembly size was approximately 671.27 Mb with a contig N50 length of 31.30 Mb. 31,654 protein-coding genes were identified from the genome. Evolutionary analysis showed that *P. major* diverged from other Lamiales species at ~62.18 Mya and experienced two rounds of WGD events. Notably, many gene families related to plant acclimation and adaptation expanded. We also found that many polyphenol biosynthesis genes showed high expression patterns in roots. Some amino acid biosynthesis genes, such as those involved in histidine synthesis, were highly induced under metal (Ni) stress that led to the accumulation of corresponding metabolites. These results suggest persuasive arguments for the global distribution of *P. major* through multiscale analysis. Decoding the *P. major* genome provides a valuable genomic resource for research on dissecting biological function, molecular evolution, taxonomy, and breeding.

## 1. Introduction

*Plantago* is a large genus within the Plantaginaceae family, including over 250 species, with broad geographic distributions in temperate and high-elevation tropical regions.^1,2^ Although the *Plantago* genus is well characterized from a taxonomic perspective, its intrageneric classification is still controversial and inadequate, especially within the subgenus *Plantago*, largely due to the plesiomorphic characters, low morphological variation, and lack of a reference genome.^1,3^ Some *Plantago* species have cosmopolitan distributions and several are used as herbal medicine, food ingredients, and in pastures, such as *Plantago major*.^4,5^

*Plantago major*, broadleaf plantain or greater plantain, is diploid (2*n* = 12), wind-pollinated, and self-compatible.^1,6^ It is a perennial herbaceous plant with a fibrous root system, a rosette of oval-shaped leaves, and several long spike inflorescences. It can be found in soils with a wide range of fertility, pH, temperature, and moisture and shows outstanding tolerance to diseases, pests, radiation, and chemical pollution.^7,8^*P. major* is native to central, northern and southwest Asia, and Europe and naturalized worldwide.^9^ It grows in various habitats, including meadows, wastelands, roadsides, and other sites of anthropogenic disturbance.^9^

To develop resistance and adaptation, plants have evolved with a series of responding mechanisms such as DNA repair, plant–pathogen interaction, and metabolic changes. Metabolic changes include synthesis and accumulation of metabolites. Such specialized metabolism may confer plants with stress resistance and involve the production of primary metabolites such as amino acids and nucleic acids and secondary metabolites such as phenolics and flavonoids.^10^ These specialized metabolites may not directly play a part in plant growth and development, but they are essential in interacting with the environment for adaptation and defense. In the case of primary metabolites, some amino acids (e.g. proline, histidine, and glutamic acid) can act either as signaling molecules or as chelators dealing with stresses such as drought, salt, and metal stresses or as precursors in the biosynthesis of secondary metabolites.^11,12^ Even more so with secondary metabolites, in general, ecological and environmental disturbance can induce their accumulation.^13,14^ Polyphenols are an excellent example of secondary metabolites elevating plant adaptation: because these molecules are involved in drought, high soil salinity, extreme temperature, UV-irradiation, nutrient deficiencies, metals, and pathogen attacks.^15^

*Plantago* contains numerous secondary metabolites, such as phenolic compounds and flavonoids.^11,16^ Some free amino acids and polyphenols can activate plant tolerance to drought and extreme temperature, defense against pathogen attack, inhibition of DNA damage, and chelation of metal ions.^15,17,18^*P. major* has the potential as a pioneer in phytoremediation of contaminated soil by accumulating metal ions (e.g. Cu, Pb, Zn, Cd, Cr, and Ni)^19,20^ that may be based on its production of certain specialized metabolites. *P. major* is also a model plant for vasculature biology study since vascular tissue can be isolated easily and intactly from mature leaves.^21^ A handy and efficient transformation method for *P. major* was also developed,^22^ which can implement functional verification *in situ*. Based on these applications, the transport of both nutrient and information molecules was well characterized, such as the transporters of sucrose,^23^ the responses to salinity,^24^ and the responses under phosphate (Pi) deficiency.^25^

To date, the chemical compounds, ecology, and population genetics of *P. major* have been widely studied.^26–28^ However, the molecular mechanisms underlying its high pollution tolerance and broad fitness in diverse environments are largely unknown. A high-quality genome assembly is necessary to address these questions. We conducted a chromosome-level genome assembly of *P. major* and found that genes accounting for the biosynthesis of specialized metabolites, such as free amino acids and polyphenols, were expanded. The genes related to polyphenol biosynthesis are more highly expressed in roots. Another expanded gene family, the histidine biosynthetic (HISN) gene family, which correlates strongly with Ni tolerance, has been characterized. The expression patterns of *PmHISN* genes provided clues for the tolerance of this species to Ni. These genomic data will provide clues for elucidating the molecular mechanism underlying the robust adaptation of *P. major* to diverse environments. The reference genome will be a valuable resource for genetic studies and improvement of Plantago, such as genome-assisted breeding of novel cultivars with low-level heavy metal ions.

## 2. Materials and methods

### 2.1 . Sequencing, genome size estimation, and assembly

**Figure 1.**
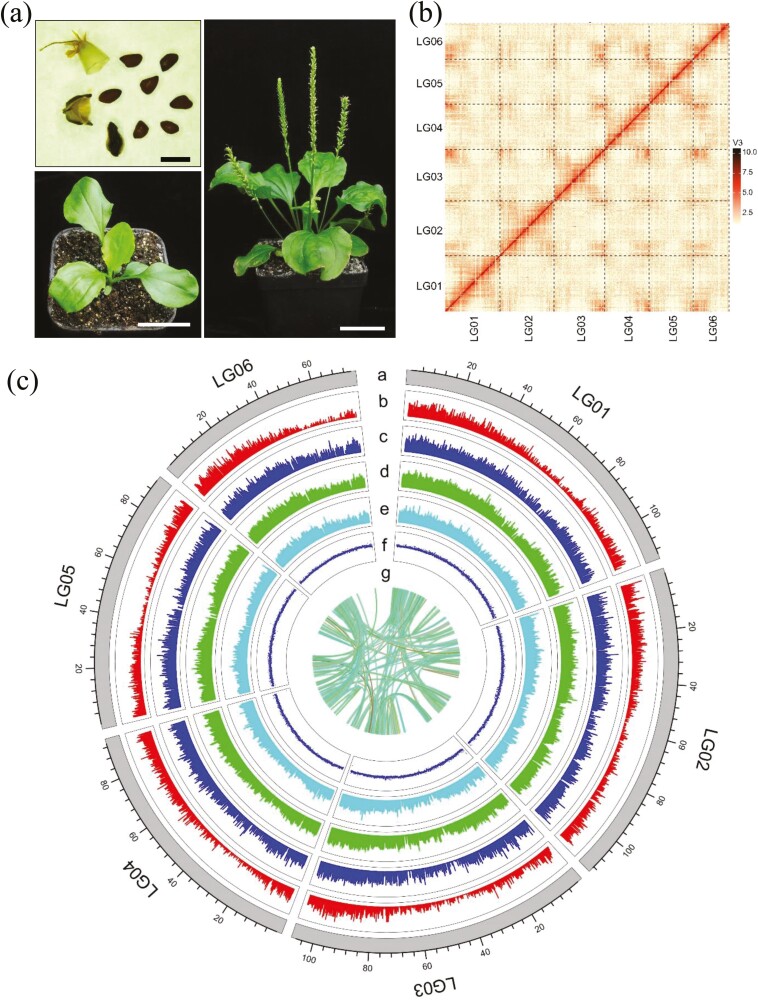
*De novo* genome assembly and annotation of *P. major*. (a) Morphological characteristics of *P. major* include seeds, seed pods, seedlings, and adult plants. Black scale: 2 mm; white scale: 4 cm. (b) Contact map of Hi-C-based intra-chromosomal interactions. (c) Genetic collinearity among six *P. major* chromosomes. (a) chromosome length; (b) gene density; (c) gene expression pattern in leaf; (d) gene expression pattern in root; (e) gene expression pattern in seed; (f) GC content; and (g) paralogous gene pairs.

One individual of *P. major* (PlanMa1, Fig. 1a) was provided by the Shennong Caotang Museum of Traditional Chinese Medicine in Guangzhou, China (113.3445 E, 23.2029 N). It is an inbred line that descended from a single seed for six generations. *P. major* and its seeds as Chinese herbs have the effect of clearing heat, diuretic and laxative. The total genomic DNA was isolated from fresh leaves using a DNA extraction kit (QIAGEN, Hilden, Germany). Three Nanopore libraries with insert sizes larger than 20 kb were constructed according to a standard protocol (Oxford Nanopore Technology, Oxford, UK), followed by single-molecule DNA sequencing. The libraries were sequenced with flow cells on the PromethION platform (Oxford Nanopore Technology, Oxford, UK). A total of 130.60 Gb (~180× of the estimated genome size) read bases were generated. Adapters and low-quality reads (*Q* ≤ 15) were removed from datasets. Before genome assembly, we estimated the genome size utilizing the *K*-mer method. The number of 17-mer sequences was counted by KmerFreq as included in SOAPdenovo package v2.04.^29^ The *P. major* genome size was estimated by the following formula: G = *K*_num_/*K*_depth_, where the *K*_num_ refers to the total number of *K*-mers, and *K*_depth_ is the most frequent peak. Sequencing data were assembled using NextDenovo v1.0 (read_cuoff = 1k and seed_cutoff = 20k, blocksize = 8g) and corrected by NextPolish v1.0.1 with default parameters.^30^ The genome was assembled using the following parameters: nextgraph_options= -n 83 -Q 6 -I 0.64 -S 0.27 -N 2 -r 0.48 -m 3.81 -C 1180183 -z 20. The quality of genome assembly was assessed by BUSCO v5.3.2.^31^ The genome assembly was assessed using next-generation sequencing. The sequence map rate is 99.02% and the coverage rate is 90.83% and the final accuracy of the genome is 99.99%.

### 2.2 Hi-C assembly

Fresh leaf material was fixed in formaldehyde to give DNA–protein bonds. The restriction enzyme *Dpn*II (New England Biolabs, Hitchin, UK) was used to digest the chromatin. The 5ʹ overhang ends were filled in with biotinylated residues. After re-ligation, DNA was sheared into ~350 bp fragments by sonication. The Hi-C library was prepared following a standard procedure and sequenced on the Illumina NovaSeq 6000 platform with PE150 mode (Illumina, San Diego, USA). A total of 903.68 million clean Hi-C paired-end reads were mapped to genome assembly using Bowtie2 v2.3.2 (-end-to-end model, parameters: --very-sensitive, -L 30).^32^ LACHESIS (CLUSTER MIN RE SITES = 100, CLUSTER MAX LINK DENSITY = 2.5, CLUSTER NONINFORMATIVE RATIO = 1.4, ORDER MIN N RES IN TRUNK = 60, ORDER MIN N RES IN SHREDS = 60) assembly was conducted to cluster Hi-C contigs into chromosome groups.^33^ The number of pseudo-chromosomes was set to six according to previous karyotyping studies.^34^ Then the genome was divided into 100 kb bins. A matrix was constructed based on the pairwise comparison by Hi-C-Pro v2.11.1,^35^ and a contact map was plotted to estimate the quality of pseudo-chromosome using the ggplot2 v3.3.6 package as implemented in R.^36^

### 2.3 *De novo* genome annotation

Repetitive elements were annotated and masked in the *P. major* final genomic assembly using *de novo* and homology-based methods. First, simple sequence repeat (SSR) sequences were identified by MISA-web.^37^ Also, LTR_FINDER,^38^ MITE-Hunter^39^ as well as RepeatModeler v1.0.11 (http://www.repeatmasker.org/RepeatModeler/) were used to construct repeat libraries. Then, libraries were combined with Repbase database (https://www.girinst.org/repbase/). Finally, the repeat regions were predicted using RepeatMasker v4.0.7 (http://repeatmasker.org/cgi-bin/WEBRepeatMasker). Also, we annotated non-coding RNAs (ncRNA) in the *P. major* genome by blasting the Pfam database (http://pfam-legacy.xfam.org/), tRNAscan-SE (http://lowelab.ucsc.edu/tRNAscan-SE/), and RNAmmer (https://services.healthtech.dtu.dk/service.php?RNAmmer-1.2).

Gene structural annotation of *P. major* was performed following three strategies: (i) *de novo* prediction performed by AUGUSTUS v3.3.3^40^ and Fgenesh v2.1^41^; (ii) homology-based annotation using GeMoMa v1.6.4^42^; and (iii) finding coding regions in transcripts by PASA v2.5.2^43^ and TransDecoder v5.5.0 (https://transdecoder.github.io/). Results were combined by EVidenceModeler v1.1.1 (http://evidencemodeler.github.io/). Then, genes containing transposable elements were removed by TransposonPSI (http://transposonpsi.sourceforge.net).

The predicted genes were further functionally annotated. Sequences queried against databases including Gene Ontology (GO) (http://geneontology.org/), KEGG (https://www.genome.jp/kegg/), KOG (ftp://ftp.ncbi.nih.gov/pub/COG/KOG/), non-redundant (NR) (ftp://ftp.ncbi.nih.gov/blast/db) and Swissprot (https://www.uniprot.org/help/downloads/) by blastp (*e*-value < 1e–10). In addition, *P. major* protein sequences were compared with protein domain annotations of all available databases using InterProScan v5.32-71.0.^44^ BUSCO v5.3.2 was used to validate the correction of gene annotations with the embryophyta_odb10 and default parameters.^31^

### 2.4 Evolutionary analysis

The genomes of 14 eudicots, including *Olea europaea, Sesamum indicum, Dorcoceras hygrometricum, Striga asiatica, Salvia splendens, Handroanthus impetiginous, Erythranthe guttata, Genlisea aurea, Mikania micrantha, Fragaria vesca, Abrus precatorius, Ipomoea nil, Ricinus communis*, and *Arabidopsis thaliana* were used in the phylogenetic analysis. A monocot genome (*Setaria viridis*) was used as an outgroup for the phylogenetic analysis. OrthoFinder v2.5.4 [57] was utilized to detect orthologous groups of the *P. major* genome using an *e*-value threshold of 1*e–*10. 209 orthogroups with a minimum of 75% of species having single-copy genes were used for phylogeny reconstruction. The protein sequences were aligned using MAFFT v7.508,^45^ after which the resulting multiple sequence alignment (MSA) datasets were converted to coding DNA sequence (CDS) format using PAL2NAL version 14.1.^46^ Sites of poor alignment quality were removed by Gblocks v 0.91b.^47^ The final dataset was generated by concatenating alignments. The phylogenetic tree was constructed by RAXML v8.2.11 with the GTR model and gamma distribution.^48^ The RelTime-ML, implemented in MEGA X software, was used for an evolutionary time estimate.^49^ Fossil records from the Timetree of Life^50^ were used to calibrate the inferred tree. The CAFÉ v5.0.0 package was used to investigate the expansion and contraction of gene families.^51^ Enrichment analysis of KEGG was performed on unique genes and expansion gene families.

The 4DTv (four-fold synonymous third-codon transversion) method was used to assess the WGD (Whole-genome duplication) event of *P. major* as well as two Lamiales (*O. europaea* and *S. indicum*). An all-to-all search was performed by blastp (*e*-value < 1e–10). Collinear regions were identified by MCscan among these three genomes.^52^ Also, the synonymous substitution rates (*Ks*) of collinear regions were calculated using CodeML in the PAML package v4.8.^53^ Paralogous gene pairs, together with the gene density, gene expression pattern (leaf, root, and seed), and GC content were visualized using the R package ‘circlize’.^54^

### 2.5 Distribution and ecological ranges

For analyzing the distribution and ecological ranges of *P. major* and three other globally distributed species (*I*. *nil, M*. *micrantha*, and *S*. *viridis*), we first obtained geo-referenced locality data for each species from the GBIF (Global Biodiversity Information Facility) (GBIF, https://www.gbif.org) in R, and the *occ_download* function in the *rgbif* package (https://CRAN.R-project.org/package=rgbif). We checked each species’ occurrence to ensure the database was representative of their distributions. For each locality, we extracted the aridity index (AI = mean annual precipitation/potential evapotranspiration; MAP/PET) using the Global-Aridity dataset (https://cgiarcsi.community/data/global-aridity-and-pet-database/). Next, we extracted soil nitrogen concentration and soil bulk density for the 0–20 cm soil depth from the World Soil Database^55^ at 1 × 1 degree resolution. To compare different ecological ranges among species, we used ANOVA and multiple comparisons (Tukey HSD) based on the mean and variance of each environmental factor across the whole range.

### 2.6 Transcriptome sequencing

The PlanMa1 plants were cultivated in pots filled with a substrate mixture of peat moss and perlite in a 3:1 ratio. The plants were maintained under constant conditions of 26°C temperature and a photoperiod of 16 h of light followed by 8 h of darkness. Total RNA of 40-day-old seedlings was extracted from three replicates of fresh leaves, roots, and seeds and treated with DNase I (QIAGEN Genomic). The RNA integrity was validated using the NanoDrop One UV–Vis spectrophotometer. Then mRNAs were enriched by Oligo (dT)-attached magnetic beads and random hexamers were used for cDNA synthesis. RNA-sequencing libraries were subsequently sequenced on the Illumina HiSeq platform with PE150 mode. Raw data were filtered by fastp v0.12.6.^56^ The FPKM (Fragments per kilobase per million mapped reads) was used to estimate the expression level of transcripts. In this study, we focus on the expression of polyphenol synthesis genes, and FPKM values were calculated by StringTie v2.1.7.^57^ Also, differential gene expression analysis was conducted using DESeq2 with fold change ≥ 2 and FDR-adjusted *P*-value ≤ 0.05.^58^

### 2.7 PmHISNs identification and expression pattern analysis

*Arabidopsis* HISN protein sequences were used as queries to perform BLASTP searches to the *P*. *major* database with the *e*-value<1e–10. Only those with *e*-value<1e–100 were kept as candidates. A phylogenetic tree was drawn by MEGA X^49^ with the Maximum Likelihood method and the bootstrap value of 1,000 using the protein sequences of AtHISNs and PmHISNs.

The *P. major* (PlanMa1) seeds were sown in soil in pots and subsequently maintained in a controlled greenhouse with 16 h light and 8 h darkness periods at 25°C. After 35 days, the experimental *P. major* seedlings were transplanted into a half-strength Hoagland solution, which was replaced on alternate days. Following an additional week, the seedlings were transferred to fresh half-strength Hoagland solutions that were supplemented with varying concentrations of NiSO_4_ (0 μM, 200 μM, and 500 μM) for a duration of 24 h. Plants were harvested and split into roots and shoots, with the root material being washed with double-distilled water to remove any residual Ni ions.

All samples intended for qRT-PCR were snap-frozen in liquid nitrogen and subsequently stored at –80°C until required. RNAs were extracted as previously mentioned and reverse-transcribed using an oligo (dT) primer in combination with SuperScript II reverse transcriptase (Vazyme). qRT-PCR was performed in the Quantagene™ q225 Detection System, using SYBR Green Master Mix reagent (Vazyme) according to the manufacturer’s instructions. *PmACTIN2* served as the internal control, and gene expression levels were determined using 2^ˉΔΔCt^ method.^59^ A list of the primers used is provided in Supplementary Table S1. The organ-specific expression patterns of *AtHISN1A* and *AtHISN1B* were obtained from the TAIR database (https://www.arabidopsis.org/).

### 2.8 Free amino acids measurement

For the analysis of free amino acids (FAA), all samples, including leaves and roots, were subjected to drying in an oven set to 80°C for a duration of 24 h. 0.50 g of each sample was extracted utilizing 25 ml of 0.01 M HCl for 30 min, at ambient temperature. Following centrifugation, 2 ml of supernatant was transferred into new tubes and combined with equal volumes of an 8% (v/v) sulfosalicylic acid solution. The resultant mixtures were centrifuged at 12,000 rcf for 5 min. Finally, the supernatant was analyzed by Amino Acid Analyzer (Sykam S433, Eresing, Germany).

## 3 Results

### 3.1. *De novo* assembly of *P. major* genome

A total of 96.66 Gb (Gigabases) Nanopore sequencing data were generated and used for further analysis (Supplementary Table S2). The genome of *P. major* consists of six pairs of chromosomes (2*x* = 12, *n* = 6), and its size is approximately 690 Mb (Megabases).^6^ In this study, the genome size was estimated to be ~701 Mb based on *K*-mer analysis (Supplementary Figure S1a), and the final assembly was 671.27 Mb with a contig N50 size of 31.30 Mb (Table 1). The longest contig reached 72.24 Mb. The quality of the genome assembly was assessed by BUSCO (Benchmarking Universal Single-Copy Orthologs).^31^ We successfully detected 95.49% of the complete BUSCO (S + D) (Supplementary Figure S1b and Table 1).

**Table 1:** Statistics for the assembly of *P. major* genome

Contig assembly		
Parameter	Length of contigs (bp)	Number of contigs
N50	31,302,600	8
N90	217,560	128
Longest contig	72,236,228	1
Total	671,270,126	615

Chromosome-level assembly		
Chromosome ID	Size (bp)	Number of contigs
LG01	113,784,451	32
LG02	112,697,239	30
LG03	106,125,141	28
LG04	93,210,706	15
LG05	91,724,435	32
LG06	74,687,028	20
Total	592,229,000	157

Assessments of genome assembly by BUSCO		
Type	Number	Percent (%)
Complete BUSCOs (C)	1,313	95.49
Complete and single-copy BUSCOs (S)	1,224	89.02
Complete and duplicated BUSCOs (D)	89	6.47
Fragmented BUSCOs (F)	5	0.36
Missing BUSCOs (M)	57	4.15
Total BUSCO groups	1,375	100

Based on Hi-C assembly with the agglomerative hierarchical clustering algorithm, 157 contigs containing 592.23 Mb Hi-C data were arranged and placed on six pseudochromosomes, representing 88.23% of total bases (Table 1). The size of chromosomes ranged from 74.69 to 113.78 Mb. A contact map was plotted to validate the correction of the Hi-C assembly; the assembled six pseudochromosomes (named LG01–LG06) corresponded to the chromosome numbers in *P. major* (*n* = 6) (Fig. 1b).

### 3.2. Annotation of *P. major* genome

Repetitive elements were annotated and masked in *P. major* before gene prediction. A total of 3.90 million SSR were detected (Supplementary Table S3). The repeat elements of the *P. major* genome were estimated to be 469.83 Mb, corresponding to 69.99% of the genomic assembly (Supplementary Fig. S1c and Supplementary Table S4). Non-coding RNAs (ncRNAs) were predicted in the genome as well (Supplementary Table S5).

Protein-coding genes were annotated by integrating *de novo* homology-based and RNA-Seq-based results. The generated consensus *P. major* gene set included 31,654 protein-coding genes, and the average length of coding DNA sequences (CDS) was 1,184.4 bp. The mapping rate of RNA-Seq reads was 99.02% and the coverage rate of annotated genes was 90.83%. Functions of 28,390 genes were annotated, corresponding to 89.69% of the predicted genes (Table 2). The quality of the annotation was assessed by BUSCO (Benchmarking Universal Single-Copy Orthologs).^31^ We successfully detected 1,350 BUSCOs in the embryophyta_odb10 database, corresponding with 98.18% of *P. major* genes (Supplementary Fig. S1b and Table 2). After gene annotation, we analyzed gene density, GC content, organ-specific gene expression patterns, and paralogous genes (Fig. 1c). There were 2048 (12.93%) pairs of tandem genes and 4685 (14.80%) collinear genes (Supplementary Tables S6 and S7).

**Table 2:** Statistics for functional annotation of *P. major* genome

Functional annotation of *Plantago major* genes		
Databases	Number of genes	Percent (%)
GO	13,803	43.6
KEGG	10,963	34.6
KOG	15,350	48.5
NR	26,789	84.6
SwissProt	22,406	70.8
Total functionally annotated	28,390	89.7
Total predicted	31,654	

Assessments of functional annotation by BUSCO		
Type	Number	Percent (%)
Complete BUSCOs (C)	1,350	98.18
Complete and single-copy BUSCOs (S)	1,247	90.69
Complete and duplicated BUSCOs (D)	103	7.49
Fragmented BUSCOs (F)	10	0.73
Missing BUSCOs (M)	15	1.09
Total BUSCO groups	1,375	100

### 3.3. Evolutionary analysis

**Figure 2.**
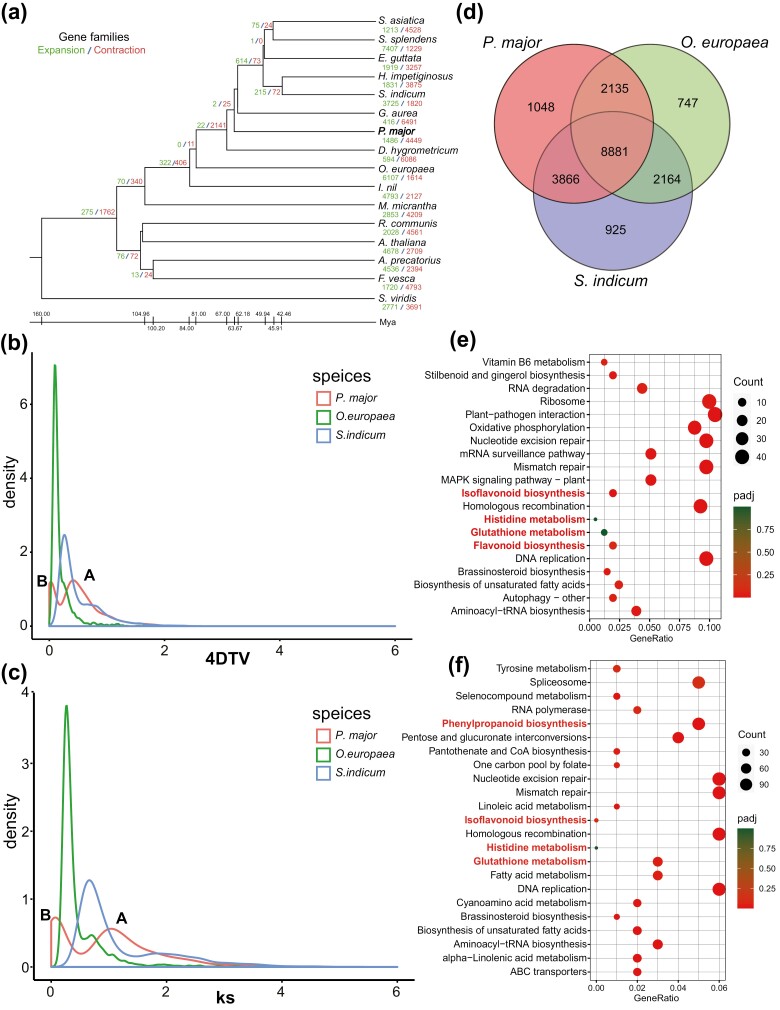
Evolutionary analysis of the *P. major* genome. (a) Phylogenetic tree of 16 angiosperms. Node and taxa labels are numbers of gene families manifesting expansion (green) and contraction (red) among 16 angiosperms. The evolutionary time scale is displayed below the tree. Distribution of 4DTV (b) and *Ks* (c) in three Lamiales. Two WGD events were observed in *P. major* (indicated as A and B). (d) Overlap of gene families in three Lamiales. (e) KEGG enrichment analysis of unique gene families in the *P. major* genome. (f) KEGG enrichment analysis of expansion gene families in the *P. major* genome.

The genome of *P. major* was compared with that of other angiosperms, including *Striga asiatica, Salvia splendens*, *Erythranthe guttata*, *Handroanthus impetiginous*, *Sesamum indicum*, *Genlisea aurea*, *Dorcoceras hygrometricum*, and *Olea europaea,* which are all Lamiales and thus phylogenetically closely related to *P. major*, and that of *Ipomoea nil, Mikania micrantha, Ricinus communis, Arabidopsis thaliana, Abrus precatorius*, and *Fragaria vesca*, which are globally widely distributed species. A monocot genome (*Setaria viridis*) was used as an outgroup in the phylogenetic analysis (Fig. 2a and Supplementary Table S8). Among the total 31,654 annotated genes in the *P. major* genome, 4,480 genes from 810 families in the *P. major* genome were species-specific compared with the other 15 species (Supplementary Table S8).

Whole-genome duplication (WGD) is a prevalent phenomenon in angiosperm plants, providing a vast source of raw genetic material for gene genesis. In this study, we investigated genome expansion in *P. major* by analyzing WGD events. We estimated 4DTv and Ks values based on paralogous gene pairs within collinear regions identified in *P. major*, *O. europaea*, and *S. indicum*. Genome-wide doubling events generate numerous homologous genes, as reflected in the Ks values, which exhibit a large number of homologous gene pairs with closely clustered Ks values, and Ks peaks correspond to the occurrence of genome-wide doubling events. A larger number of gene pairs with 4DTV presence suggests greater genomic diversity or an increased number of redundant genes, possibly indicating species differentiation or ongoing genome duplication. Intra-genome collinearity analysis revealed sharp peaks in both 4DTV and Ks, confirming the occurrence of WGD events in *P. major*, *O. europaea*, and *S. indicum* (Fig. 2b and c). Furthermore, *P. major* 4DTV results exhibited two peaks, signifying two WGD events: one occurred during the early evolutionary stage of Lamiales (Peak A, 4DTV ≈ 0.55 and Ks ≈ 1.55), and the other was a more recent genome duplication, potentially occurring within Plantaginaceae (Peak B, 4DTV ≈ 0.35 and Ks ≈ 0.07) (Fig. 2b and c). In contrast, *O. europaea* and *S. indicum* had only one pronounced WGD event.

### 3.4. Unique and expanded genes enriched in metabolite biosynthesis and defense in *P. major*

A comparison of gene families was made among *P. major, O. europaea*, and *S. indicum* (Fig. 2d). The three species shared 8,881 out of the 15,930 orthologous gene families. There were 1,048 unique gene families in *P. major*, which shared more gene families with *S. indicum* (3,866) than with *O. europaea* (2,135) (Fig. 2d). KEGG enrichment analysis indicated unique families enriched in primary metabolite pathways such as histidine metabolism (map00340) and secondary metabolite pathways such as the phenylpropanoid biosynthesis pathway (map00940), isoflavonoid and flavonoid biosynthesis pathways (map00943 and map00941), and glutathione metabolism (map00480). Free histidine (His) can chelate Ni in plants and is responsible for nickel transport and tolerance.^60^ Phenylpropanoids, isoflavonoids, and flavonoids can improve plants’ tolerance to environmental stresses.^14^ Glutathione is essential for PCs (phytochelatins) synthesis, a principal class of metal chelators (Yadav 2010). Unique genes in DNA repair (nucleotide excision repair and mismatch repair, i.e. ko03420 and ko03430) and plant defense activities (plant–pathogen interaction, i.e. ko04626) were also enriched in *P. major* (Fig. 2e).

Compared with other angiosperms, 1,486/4,449 gene families manifested expanded/contracted patterns in *P. major*. The KEGG enrichment analysis of expansion showed similar results for unique families. Some expanded gene families were related to the biosynthesis of amino acids (map01230), histidine metabolism (map00340), the phenylpropanoid biosynthesis pathway (map00940), isoflavonoid and flavonoid biosynthesis pathways (map00943 and map00941), and glutathione metabolism (map00480) (Fig. 2f). These results indicate that those resistance genes retained after WGD events may allow *P. major* to exhibit a global distribution and adaptations, and the specialized metabolites may explain *P. major*’s repertoire of habitat types and environmental conditions.

### 3.5. Adaptation strategy of *P. major*

**Figure 3.**
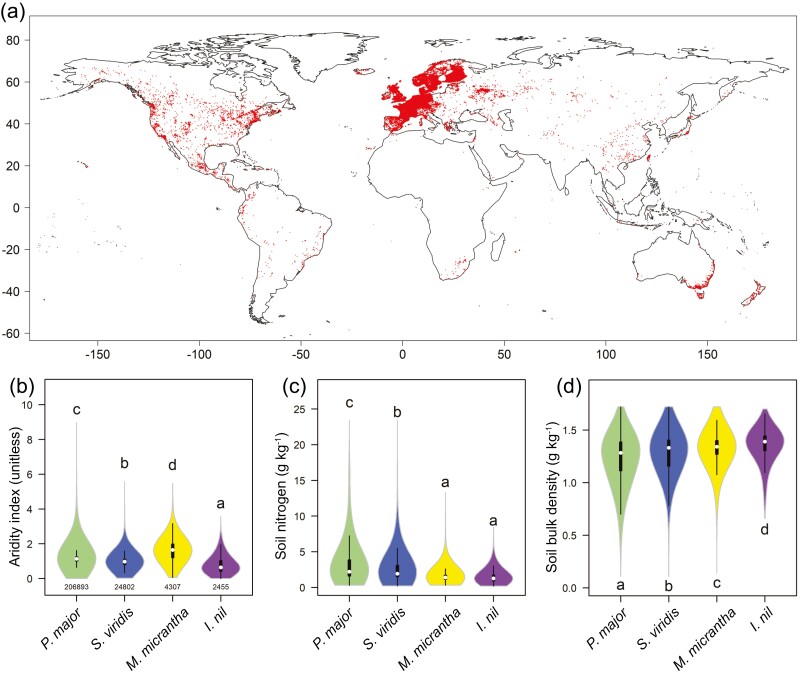
Global distribution of *P. major* and ecological range comparisons with three other globally distributed herbaceous species. (a) Distribution localities of *P. major*. Red dots indicated regions of *P. major* distribution and the deeper of the color, the more of the *P. major*. (b–d) Comparisons of environmental factors among four globally distributed species: (b) aridity index (indicating water stress), (c) soil nitrogen concentration (indicating soil fertility), and (d) soil bulk density (indicating resistance to trampling). Sample sizes for each species are under each violin in (b). Data are based on climatic variables and soil properties across global distribution localities of each species. The violin shows data distribution and niche breadth of each species, while the black boxplot inside each violin indicates the median (white dot), first and third quartiles (upper and lower limits of the box), and the interquartile range (whiskers). Difference letters on top of each violin indicate significant differences based on multiple comparisons (Tukey HSD).

*Plantago major* has widely naturalized throughout much of the world (Fig. 3a). Compared with three globally distributed species (*I*. *nil*, *M*. *micrantha*, and *S*. *viridis*), *P. major* occupies much larger ranges of climatic conditions and soil environments (the widest interquartile ranges in Fig. 3b–d). Specifically, *P. major* grows over a wide climatic range, but moderately in arid environments (intermediate aridity index values, Fig. 3b) and acidic or infertile soils (the most significant values of soil nitrogen concentration and the lowest values of soil bulk density; Fig. 3c and d). The resistance genes mentioned above may contribute to *P. major*’s adaptation to harsh conditions. Given its wide adaptation, *P*. *major* can be used as a pioneer plant in new assarts or disturbed lands.

### 3.6. High expression of polyphenol synthesis genes contributes to *P. major*’s global distribution

Polyphenols have properties of defense against biotic and abiotic stresses such as pathogen attacks, oxidants, and ultraviolet radiation.^61^ We identified genes in *P. major* involved in polyphenol synthesis (Supplementary Fig. S2). Most gene families involved in polyphenol metabolism were expanded, for example, cinnamoyl-CoA reductase (EC: 1.2.1.44) and 4-coumarate-CoA ligase (EC: 6.2.1.12).

**Figure 4.**
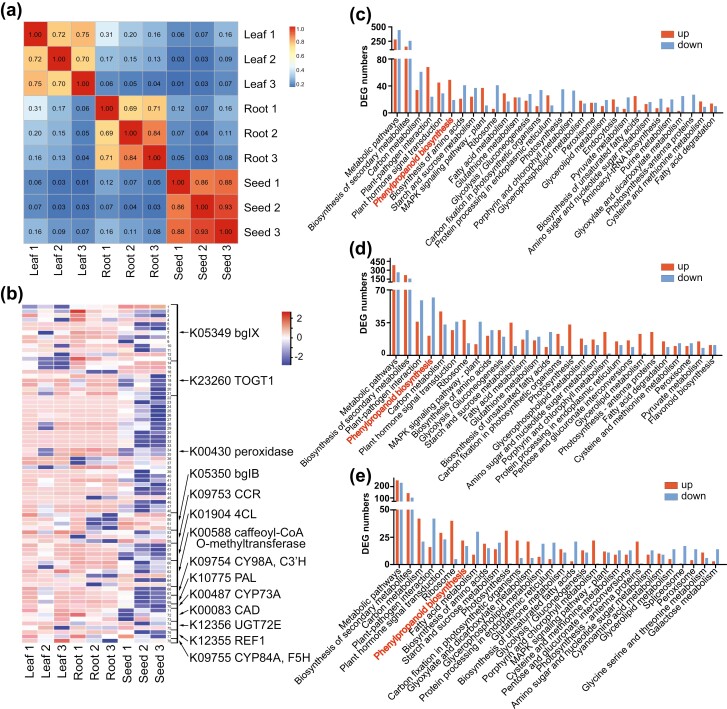
Expression of phenylpropanoid biosynthesis genes. (a) Transcriptome correlations (Pearson’s correlation coefficients) across samples for each tissue for all annotated genes. (b) The heatmap of genes related to phenylpropanoid biosynthesis. Blue cells denote down-regulated DEGs, and red cells denote up-regulated DEGs. (c) Protein classes of DEGs and KEGG pathways enriched by up- (red) and down- (blue) regulated genes between leaves and roots. (d) Protein classes of DEGs and KEGG pathways between roots and seeds. (e) Protein classes of DEGs and KEGG pathways between leaves and seeds.

To assess differences in gene expression patterns of phenylpropanoid biosynthesis genes among organs, we analyzed 78 differentially expressed genes (DEGs). The correlation of the expression patterns in different organs was relatively low, while replicates of the same organ showed a similar expression pattern (Fig. 4a). Moreover, the heatmap of the DEGs indicated that leaves, seeds, and roots elegantly exhibited quite different transcriptome profiles (Fig. 4b). Most genes showed a higher expression level in roots than in leaves and seeds, except for the K05350 bglB. Organ-specific analysis of DEG indicated that the expression level of phenylpropanoid biosynthesis genes was significantly higher in roots than in leaves and seeds (Fig. 4c–e). The numbers of up- and down-regulated genes in roots and leaves were 49 and 19, respectively. For root/seed and leaf/seed comparisons, the numbers of up- and down-regulated genes were 62/21 and 22/17. The results of our analysis suggest that the expression level of phenylpropanoid biosynthesis genes is significantly higher in roots compared to leaves and seeds in *P. major*. Our findings indicate that the plant would activate polyphenol synthesis in its roots, which may contribute to its global distribution.

### 3.7. Highly expressed histidine biosynthesis genes confer *P. major* high tolerance to Ni stress

*Plantago major* is a pioneer known to grow in polluted areas with high concentrations of metals, such as Ni.^20^ As free His is a detoxicant of Ni in plants^60^ and the genes related to His metabolism were expanded in *P. major*, we further explored the His biosynthesis (HISN) gene family.

**Figure 5.**
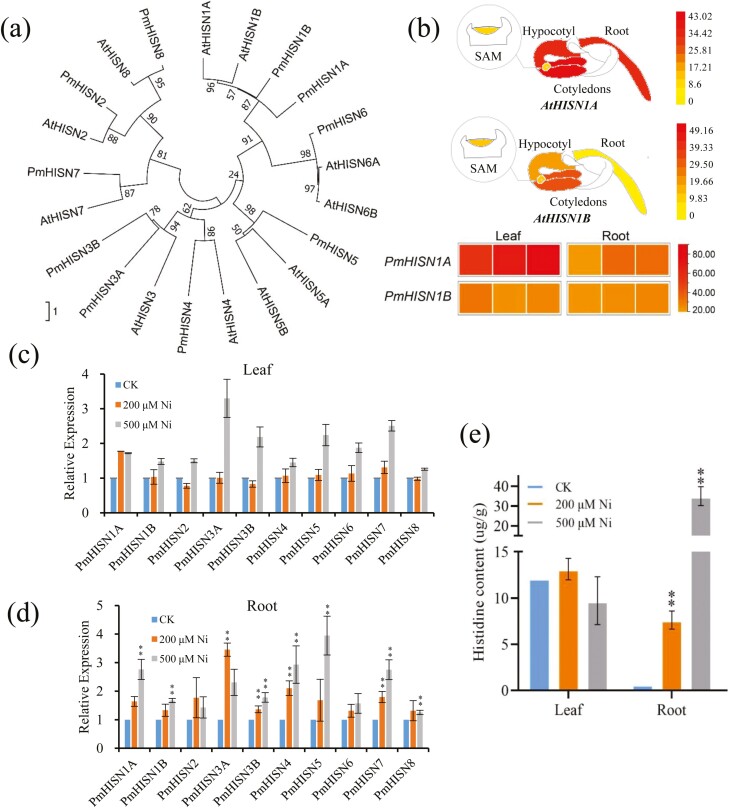
The identification and expression patterns of PmHISNs and their response to Ni treatment. (a) The phylogenetic tree of *AtHISNs* and *PmHISNs*. (b) The expression patterns of *AtHISN1A/B* and *PmHISN1A/B* in leaves and roots; (c and d) The expression patterns of *PmHISNs* under Ni treatments in *Plantago major* leaves and roots, respectively. (e) The histidine content in *P. major* leaves and roots under Ni treatments respectively. CK: the control without Ni treatment. Data represent the mean ± SD of three biological replicates. The student’s *t*-test is evaluated respect to the control *P < 0.05, **P < 0.01.

Ten *PmHISNs* were identified based on Arabidopsis orthologs. All *PmHISNs* were designated according to *AtHISNs*, including *PmHISN1A* (*LG06.1131*), *PmHISN1B* (*LG05.986*), *PmHISN2* (*LG04.4921*), *PmHISN3A* (*LG01.5679*), *PmHISN3B* (*LG03.4142*), *PmHISN4* (*LG06.3238*), *PmHISN5* (*LG01.4174*), *PmHISN6* (*LG02.3515*), *PmHISN7* (*LG01.4789*), and *PmHISN8* (*LG02.1456*) (Fig. 5a and Supplementary Table S9).

HISN1A and HISN1B catalyze the first and also the rate-limiting step in the His biosynthetic pathway, and over-expression of HISN1A/B lead to significant accumulation of His.^62^ When compared with Arabidopsis, the expression levels of *PmHISN1A* and *PmHISN1B* were much higher than that of Arabidopsis homologs (Fig. 5b). More importantly, *AtHISN1B* was barely expressed in roots, while *PmHISN1B* was expressed at similarly high levels in leaves and roots (Fig. 5b). To explain the difference, we explored the gene and protein structures. *PmHISN1A/B* share similar gene structures with *AtHISN1A/B* which were organized into 11 exons and 10 introns. (Supplementary Fig. S3a). AtHISN1A/B and PmHISN1A/B proteins all encompass HisG and HisG_C (HisG, C-terminal) domains, whereby the HisG is ATP phosphoribosyltransferase participating in histidine metabolism (Supplementary Fig. S3b). But the prominent promoters of *PmHISN1B* were many more than *AtHISN1B* (Supplementary Fig. S3c). The accumulation of His is positively correlated with the mRNA levels of *HISN1A* and *HISN1B*, which suggests that in comparison with Arabidopsis, a higher concentration of His and greater Ni tolerance is expected in *P. major*. In addition, more HISN genes in related species (within Lamiales) of *P. major* partially explain its high Ni resistance (Supplementary Fig. S4).

To analyze *PmHISNs*’ response to Ni stress, we treated *P. major* with different concentrations of Ni^2+^ (200 µM and 500 µM NiSO_4_ in half-strength Hoagland solution). Almost all the *PmHISNs* were induced by the Ni treatment, and a higher concentration of Ni led to higher *PmHISNs* expression (Fig. 5b and c). Moreover, since the Ni uptake occurs in the roots, the *PmHISNs* in root were more significantly induced. To confirm whether the concentration of His was influenced, we measured the levels of free amino acids. Although the concentrations of His in the leaves remained about the same, the His concentrations in roots were significantly increased (Fig. 5e). This result suggests that Ni can activate the His biosynthetic pathway in *P. major* and promote His synthesis and subsequently mediate Ni chelation. Based on these results, we surmise that the expanded genes and shifted expression patterns related to His metabolism also allowed *P. major* to exhibit a global distribution.

## 4. Discussion

Due to a dearth of valid taxonomic characters, the intrageneric classification of the genus *Plantago* is controversial and inadequate. Several morphological features, including trichomes and seeds, as well as chemotaxonomic analyses,^63–65^ have been employed in attempts to identify and classify the species. However, none of these methods have yielded a conclusive result that is considered satisfactory, not even with 91 mainly morphological and embryological characters.^66^ Recently, high-throughput sequencing was applied to update the taxonomy of *Plantago*.^1^ Nevertheless, owing to the paucity of the reference genome, they just assembled the sequencing reads to the only published plastome of *P. media* L.^67^ As our manuscript was nearing completion, the genome of *Plantago ovata* was published, highlighting the increasing recognition of the significance of *Plantago* species in genomic research.^68^ Research on the genome of herbal medicines is becoming increasingly popular. The rapid development of next-generation sequencing and chromosome-level assembly technologies makes it possible to produce *de novo* genome assemblies.^69^ In this study, long reads of the *P. major* genome were generated by Nanopore sequencing, and a well-resolved genome was assembled by Hi-C technology. The final assembled contigs comprised six chromosomes, corresponding to 88% of the assembly. The 671.27 Mb *P. major* genome included 70% repeat sequences and 31,654 genes. The genome size and gene number of *P. major* are at a medium level among all compared species (Supplementary Table S8).

The genome assembly presented in this study represents a robust resource for clarifying the taxonomic relationships within the genus *Plantago*. Although previous research in the genus has relied heavily on molecular phylogenetic analyses based on ITS (Internal transcribed spacer),^70–73^ chloroplast,^73,74^ and mitochondrial^73^ makers or sequences, these methods have proven to be insufficient. ITS sequences are typically shorter than 500 bp and therefore possess a limited number of informative variants. In addition, ITS copies within the genome can exhibit high homogeneity due to concerted evolution,^75^ a limitation that also applies to chloroplast and mitochondrial markers. The use of ITS, chloroplast, and mitochondrial sequences in taxonomic studies is limited by several factors. As mentioned previously, these molecular markers can be subject to limited variation due to factors such as maternal inheritance and selective sweeps, respectively. These limitations can hinder the ability of these molecular markers to accurately resolve taxonomic relationships, particularly in cases of recent or rapid speciation events. Consequently, genomic data offers a more comprehensive approach that can overcome these limitations and provide a more accurate and detailed understanding of the taxonomic relationships among species. In summary, the genomic data assembled in this study offers a powerful tool for accurately resolving the taxonomic relationships within Plantago.

The genome of *P. major* was compared with that of other Lamiales, a species-rich and highly diverse order.^76^ The genome size and gene number vary widely among Lamiales; while the carnivorous plant *G. aurea* has a 43.36 Mb genome with 17,685 genes, the genome size and gene number are much larger in *D. hygrometricum* (1,521.36 Mb and 47,778 genes) (Supplementary Table S8). Multiple polyploidization events occurred during the evolution of Lamiales.^77^ In this study, both 4DTV and Ks results revealed two rounds of WGD events in *P. major*: one occurred early in the evolution of Lamiales, and the other more recently (Fig. 2). WGD events lead to a rapid increase in the genome size and expansion of gene families.^78^ Gene duplication/expansion may enhance plant disease resistance and adaptation to stress.^78^ In this case, gene expansion following WGD enriched gene families associated with adaptation to stress (e.g. phenylpropanoid biosynthesis, isoflavonoid and flavonoid biosynthesis, glutathione metabolism, histidine metabolism, nucleotide excision repair, and plant–pathogen interactions).

*Plantago* species synthesize multiple polyphenols, for example, lignin, iridoid, and caffeoyl phenylethanoid glucosides.^11,63,79^ Polyphenols confer plant tolerance to biotic and abiotic stresses such as pathogen attacks, oxidants, and ultraviolet radiation.^61,80^ As a result, polyphenols enhance the survival of plants in various environments.^81^ In this study, we identified genes involved in polyphenol synthesis (Fig. 4). Most gene families involved in polyphenol synthesis were expanded, for example, cinnamoyl-CoA reductase (EC:1.2.1.44) and 4-coumarate-CoA ligase (EC:6.2.1.12). We also investigated the expression pattern of polyphenol synthesis genes in different organs of *P. major* (Fig. 4). The expression levels of phenylpropanoid biosynthesis genes were significantly higher in roots than in aerial parts, indicating high resistance to belowground biotic and abiotic stresses. Corresponding with the above adaptive mechanisms, we noted the wide ecological distribution of *P. major* in climatic and edaphic conditions (especially drought and soil infertility; Fig. 3). A previous study indicated that polyphenol-rich plants are adapted to arid and infertile habitats; polyphenols affect root growth and reduce the toxic effects of metal ions.^82^

It is our conjecture that, unlike inter-species disparities in expression, when both *HISN1A* and *HISN1B* genes are expressed at high levels simultaneously, this can result in enhancing *P. major* Ni tolerance. One possible explanation would be that the prominent promoters of *PmHISN1B*, such as CAAT-box, TATA-box, MYB, and MYC, were many more than *AtHISN1B*, and the total number of identified promoters before *PmHISN1B* was also more than that of *AtHISN1B* (Supplementary Fig. S3c). Ni treatment induced the His biosynthesis pathway and enhanced the concentrations of some stress-related free amino acids, such as glycine, arginine, serine, leucine, lysine, and isoleucine (Supplementary Fig. S3d). Additionally, evidence is accumulating that *P. major* is a suitable species for phytoremediation of metal-polluted soils contaminated by Cu, Fe, Pb, and even radioactive U.^83,84^ To gain further comprehension regarding the function of HISNs, a comparative analysis was conducted among HISNs obtained from 16 distinct species, including those that are considered related (within Lamiales). The results of this analysis revealed that the related species tend to possess a greater number of HISN genes, particularly with respect to HISN1, which serves as the enzyme responsible for catalyzing both the initial and rate-limiting step in the His biosynthesis pathway (Supplementary Fig. S4). It should be noted that under no circumstances should *P. major* that has been cultivated within contaminated regions be employed for medicinal purposes.

In conclusion, we have successfully assembled a high-quality and chromosome-level genome of the *P. major*, and have provided an annotation for the same. This genome can serve as a reference for the investigation of gene functions, genome evolution of Lamiales, and *Plantago* taxonomy. Based on our analysis, we determined that *P. major* diverged from other Lamiales species at ~62.18 Mya and underwent two distinct rounds of WGD events. Furthermore, we observed an expansion in the genes responsible for secondary metabolism, with polyphenol biosynthesis and amino acid biosynthesis genes being significantly expressed. Notably, we observed a strong induction of His synthesis as a consequence of Ni exposure. These results may serve to explain the global distribution of *P. major*. We suggest that *P. major* can be used as a pioneer plant in a harsh environment, as well as for phytoremediation of metals.

## Supplementary Data

Supplementary data are available at *DNARES* online.

**Supplementary Figure S1.** Genome assessment and annotation of *P. major*. (a) Frequency distribution of 17-mer. *K*_num_ = 46,277,522,193 and *K*_depth_ = 66. (b) The assessment of genome assembly (upper) and functional annotation (lower) by BUSCO (c) Distribution of transposable elements. (d) The Venn diagram of gene functional annotation by GO, KEGG, KOG, NR, and SwissProt.

**Supplementary Figure S2.** The genes of the phenylpropanoid biosynthesis pathway identified in *P. major*. Expanded gene families are highlighted in yellow.

**Supplementary Figure S3.** *HISN1A/B* are largely conserved between Arabidopsis and *P. major*, but show different expression patterns. (a) The expression patterns of *AtHISN1A/B* and *PmHISN1A/B* in leaves and roots. (b) *AtHISN1A/B* and *PmHISN1A/B* gene structures. Blue blocks indicated the exons, and black lines indicated introns. (c) Domains contained in AtHISN1A/B and PmHISN1A/B. HisG is the ATP phosphoribosyltransferase domain; HisG_C corresponds to the ATP phosphoribosyltransferase, C-terminal domain. (d) The prominent domains of *AtHISN1A/B* and *PmHISN1A/B* promoters identified by plantCARE. (e)The contents of some stress-related free amino acids. Gly, Glycine; Arg, Arginine; Ser, Serine; Leu, Leucine; Lys, Lysine; Iso, Isoleucine. Data represent the mean±SD of three biological replicates.

**Supplementary Figure S4.** The summary of HISNs in 16 species. The branches of Lamiales species are indicated as red. The evolutionary time scale is displayed at the top of tree.

dsad013_suppl_Supplementary_Figure_S1

dsad013_suppl_Supplementary_Figure_S2

dsad013_suppl_Supplementary_Figure_S3

dsad013_suppl_Supplementary_Figure_S4

dsad013_suppl_Supplementary_Tables

## Data Availability

The *P. major* genome assembly was submitted to NCBI GenBank (accession number JAIFAC000000000). The raw sequencing reads and Hi-C data were deposited in the NCBI Sequence Read Archive (SRA) under the BioSample SAMN20255407. Also, nine files of transcriptome raw reads were deposited in SRA under the BioSample SAMN20959326—SAMN20959334. The annotation file is available at figshare (https://figshare.com/articles/dataset/Plantago_major_evm_gff/15149097).
