# Supplementary material for: Genome assembly of the pioneer species Plantago major L. (Plantaginaceae) provides insight into its global distribution and adaptation to metal-contaminated soil
Source: DNA Res. 2023 May 25;30(4):dsad013. doi: 10.1093/dnares/dsad013 (PMC10254747; doi:10.1093/dnares/dsad013)
Supplement: dsad013_suppl_Supplementary_Figure_S1 [file dsad013_suppl_supplementary_figure_s1.pptx]

## Slide 1
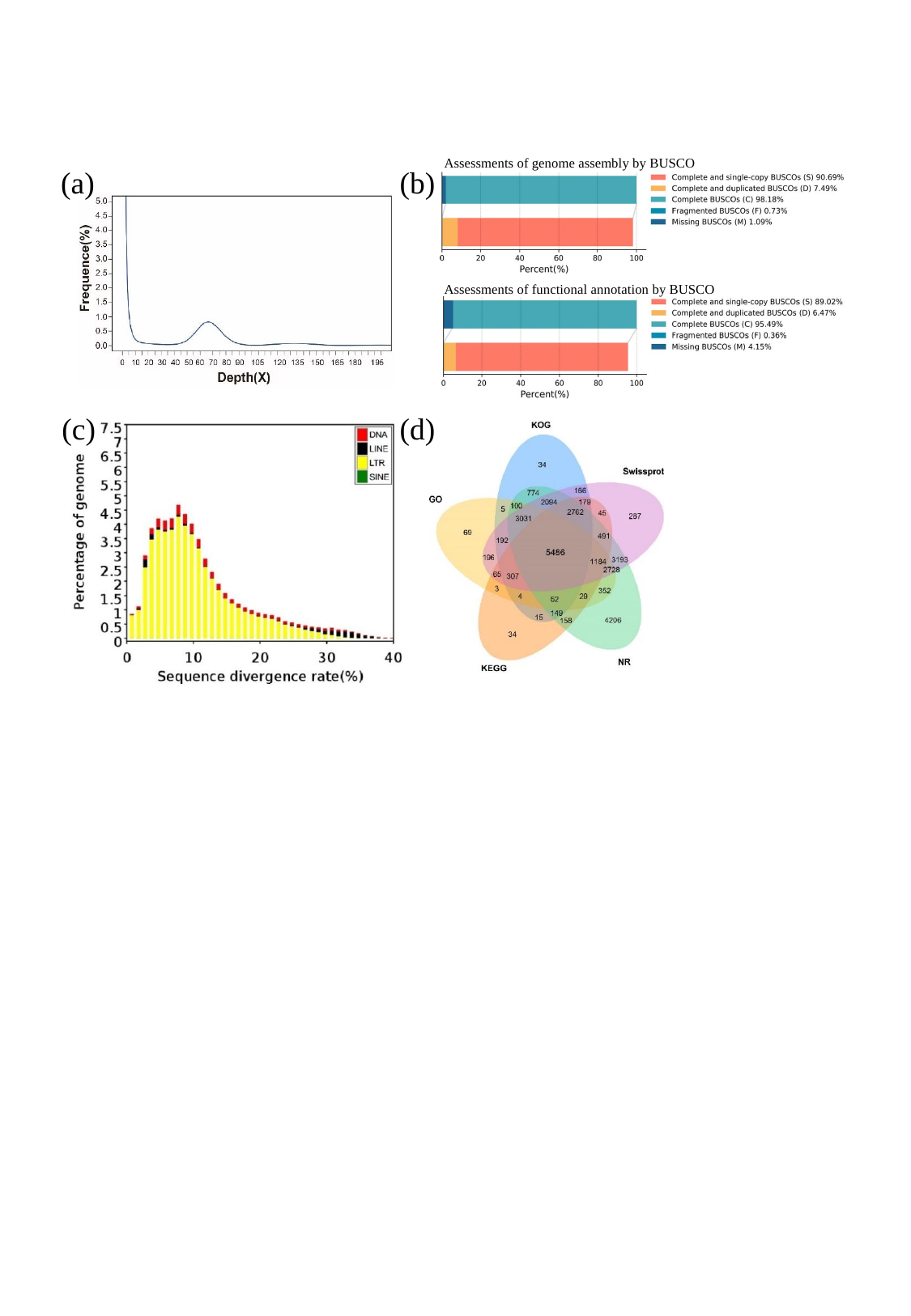

Assessments of genome assembly by BUSCO
(a)
(b)
Assessments of functional annotation by BUSCO
(c)
(d)
