# Supplementary figures and images for: Genome assembly of the pioneer species Plantago major L. (Plantaginaceae) provides insight into its global distribution and adaptation to metal-contaminated soil
Source: DNA Res. 2023 May 25;30(4):dsad013. doi: 10.1093/dnares/dsad013 (PMC10254747; doi:10.1093/dnares/dsad013)

## Slide 1
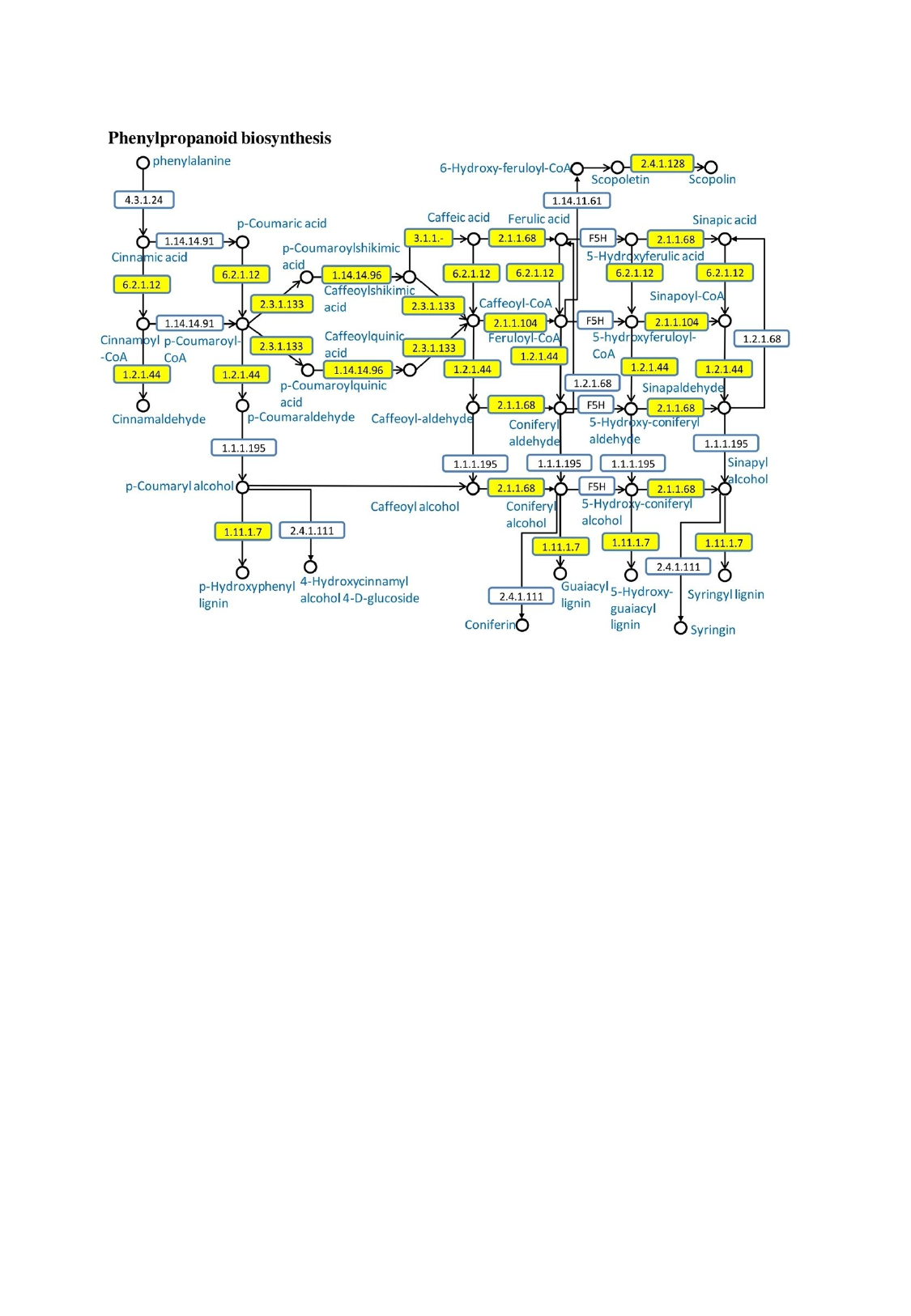

Supplement: dsad013_suppl_Supplementary_Figure_S2 [file dsad013_suppl_supplementary_figure_s2.pptx]

## Slide 1
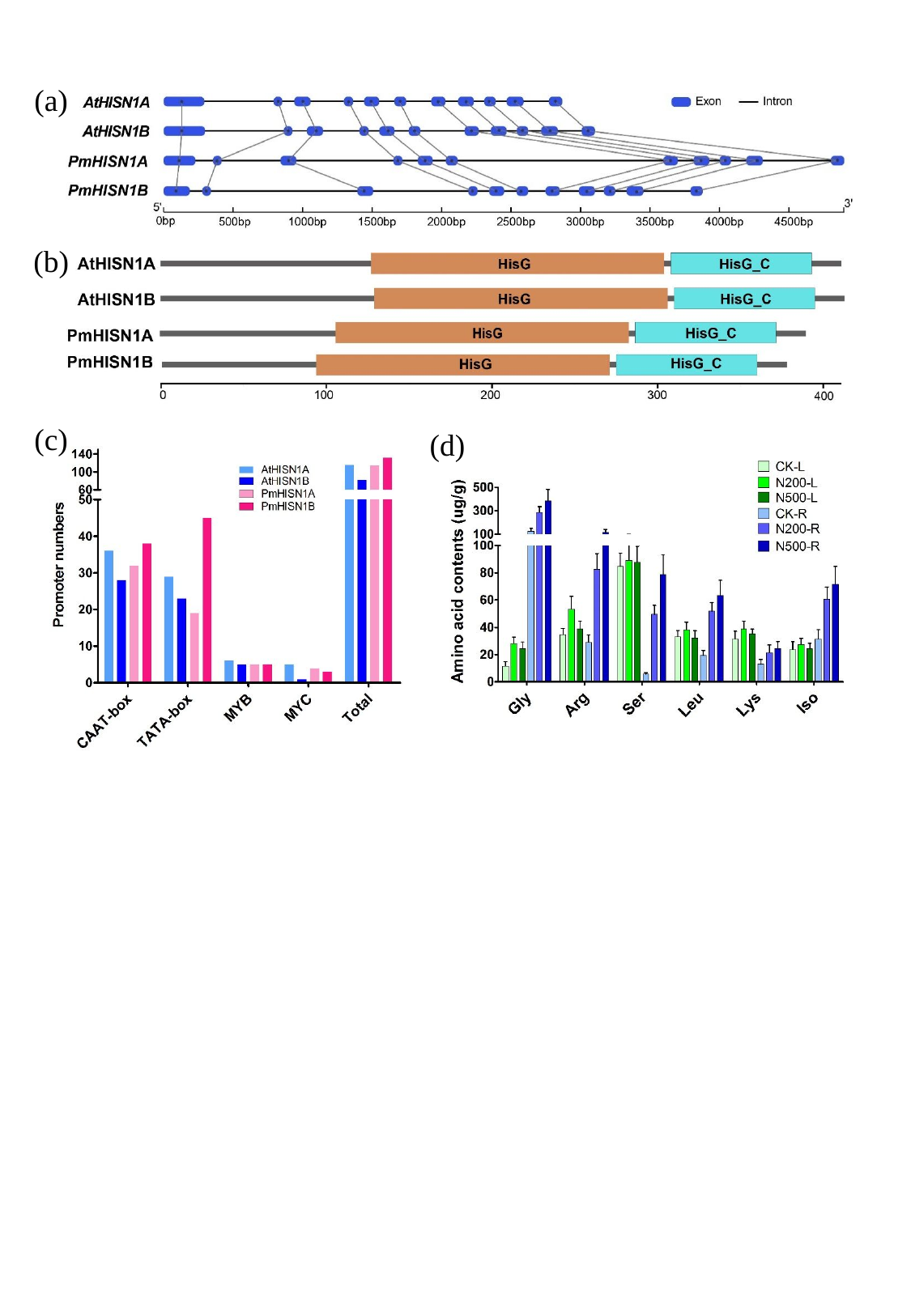

(a)
(b)
(c)
(d)

Supplement: dsad013_suppl_Supplementary_Figure_S3 [file dsad013_suppl_supplementary_figure_s3.pptx]

## Slide 1
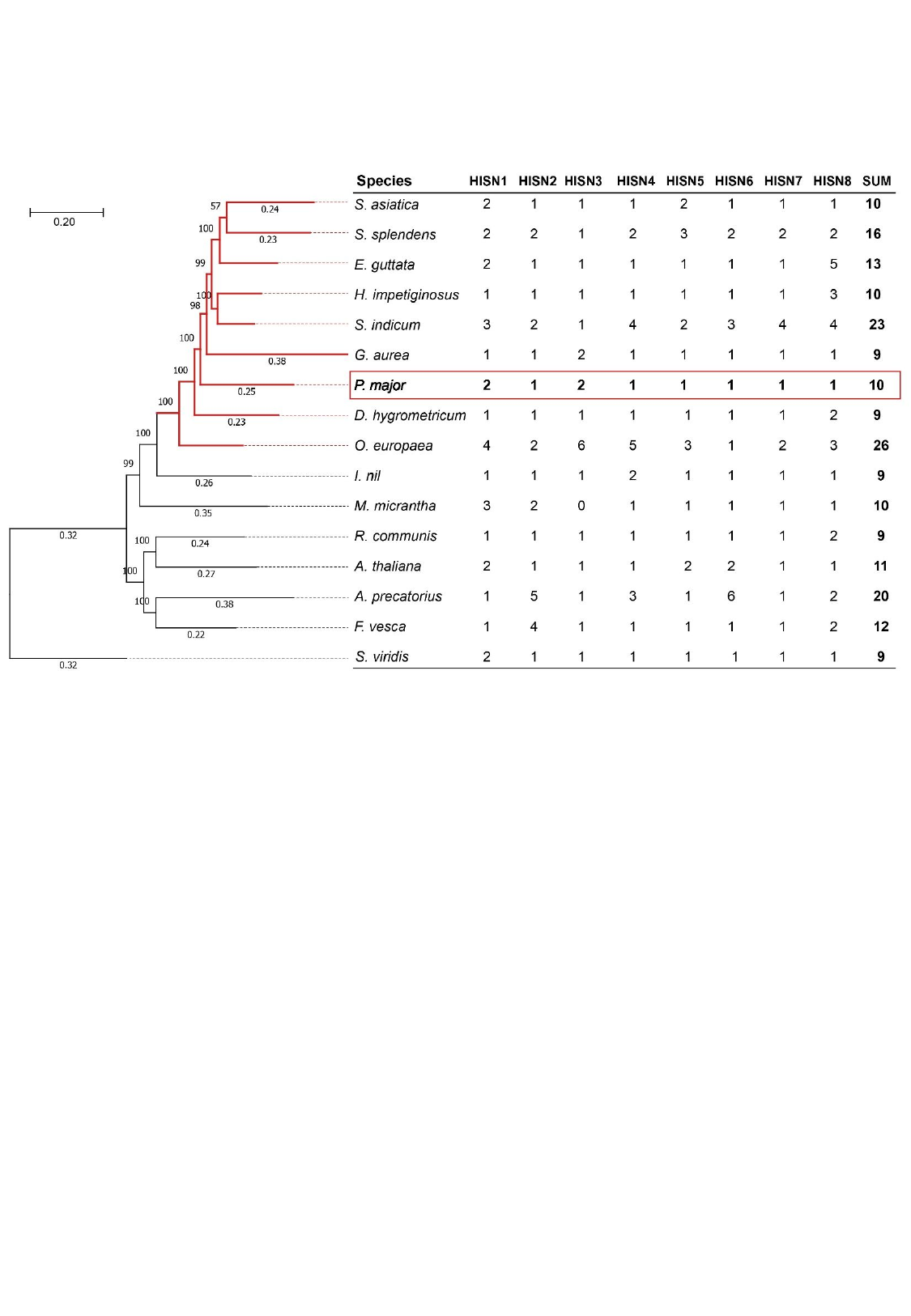

Supplement: dsad013_suppl_Supplementary_Figure_S4 [file dsad013_suppl_supplementary_figure_s4.pptx]
